# The importance of regulation (EU) 2017/746 for quality control in medical laboratories

**DOI:** 10.11613/BM.2022.010301

**Published:** 2021-12-15

**Authors:** Cristiano Ialongo, Maria Sapio, Leda Elisabetta Antetomaso, Antonio Angeloni

**Affiliations:** Department of Experimental Medicine, Policlinico Umberto I, ‘Sapienza’ University, Rome, Italy

**Keywords:** quality control, traceability, uncertainty

The guideline C24 (now in its 4th edition) issued by the Clinical and Laboratory Standards Institute (CLSI) recommends that for purchased quality control (QC), the laboratory should never use the manufacturer’s declared target (T) value but that identified by testing the product at least ten times with each new batch (*1*). Of course, verifying T is quite different from estimating the variability of the analytical process, albeit both are used for the statistical process control (SPC) since T should be the production target of the QC material on which the manufacturer has complete control.

If we consider that the reasons for the purchase of manufactured QC are facilitating the management of SPC in routine, reduce the costs and time required for in-house production, and benefit from an industrial process that ensures tighter control over the reliability of the product, then untrusting T seems paradoxical. Why should the customer pay additional costs to take advantage of a product whose benefits he already pays for?

With the European Union (EU) Regulation 2017/746 on *in vitro* diagnostics medical devices, the customer paradox seems to be bound to an end as metrological traceability (MT) becomes mandatory “where the performance of the devices depends upon calibrators and/or control materials” (see Annex I, Chapter II, section 9.3) (*2*). In fact, as it reads, “the instruction for use shall contain […] information regarding maximum (self-allowed) batch to batch variation provided with relevant figures and units of measure” of values assigned to calibrators and control materials (Annex I, Chapter III, section 20.4.1.u) (*2*). In plain words, any QC material marketed by May 2022 shall not require customer’s verification because provided with all the information to make it trustable.

Actually, a manufactured product depends on a specification that reflects some requirements of the customer. In the case of QC, specification is the quantity value of the substance in the patient’s specimen to which the laboratory needs to control the assay. As we stated earlier, this value should correspond to T. In real practice, the QC manufacturer always lays some tolerance ∆T on the specification in order to make the productive process economically advantageous (*e.g.,* to increase the shelf stability of the product). Thus, any batch can be released if T rests within T ± ∆T.

An issue with ∆T is that it tends to be somehow “permissive” to the manufacturer, and thus quite loose. Since ∆T is the “maximum (self-allowed) batch to batch variation” mentioned by EU Directive, one shall wonder whether the EU Regulation could be actually able to give an end to the customer paradox. In order to understand this, let’s suppose that an ideal automated analyser was meant to manage autonomously the SPC, and to this end, it was also able to acquire automatically the datum T to set its own Shewhart control chart (SCC). If the MT imposes to provide uncertainty on T, say u^2^(T), then based on what was stated insofar one should expect ∆T to be responsible for the largest contribution. Hence, could we run SPC without verifying T if the manufacturer provided ∆T? The answer can come from assessing the impact of ∆T in the typical (SCC).

In our example, consider a generic QC with T = 2.00 mg/L that manufacturer provides with 10% tolerance in its certificate, say ∆T = 0.01T = 0.20 mg/L. Considering that: a) T can only range within ±∆T, b) any value within ∆T is equally good for marketing, and c) the most probable T is what production aims for or ±∆T = 0, then we can calculate as follows (*3*):



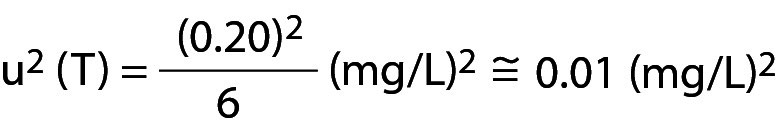



For SCC with individual measurements, robust estimates of the control limits (CL) are given by (*4*):



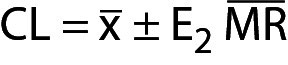



where x is the centreline value, E_2_ is a constant and MR is the mobile range. Thus posing x = T, MR = 0.21 mg/L (from a previous experiment to set up SCC) and E_2_ = 2.66 (corresponding to 3SD) it yields:



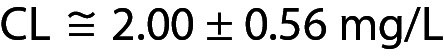



The uncertainty of CL is thus:



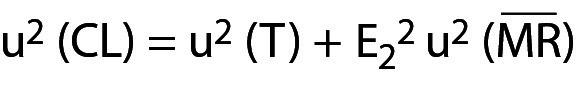



where u^2^ (MR) is derived from the standard error of MR. Since u^2^ (MR) << 0.01 (mg/L)^2^ substituting it yields:



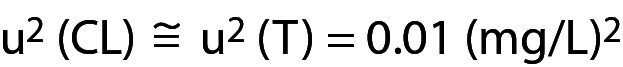



The expanded uncertainty of CL with 95% coverage is thus:



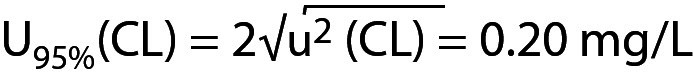



To find the critical fences on CL it is possible to add the half-width of U_(95%)_ (CL) as follows (*5*):



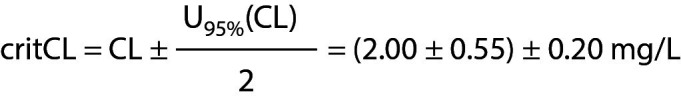



To calculate the inner and outer critCL the following are used:



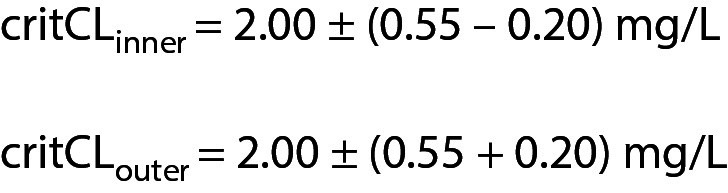



The critical fences on the centreline is calculated as following:



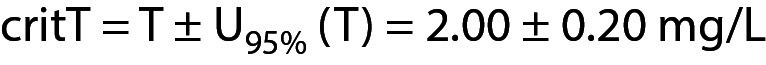



Thus, trusting T ± ∆T has two consequences. First, *per* Eq. 9, the centreline turns into a band within which all the values are equally good as T. Consequently, alarms due to repeated sequences, *e.g.,* 4 consecutive values on the same side of T at 1SD (4:1s), are less likely to be detected. Second, *per* Eq. 7 that shifts CL, the type I error (α) or probability of false positive chosen for the SCC actually changes (*i.e.,* outer critCL deflates it and the inner critCL inflates it). Likewise, also type II error (β) or probability of false negative changes. Hence, it really seems that the customer paradox has no solution, since trusting the manufacturer’s T would mean compromising with SCC performance while doing otherwise would not pay off the efforts required by the EU Regulation. Is there a way?

If the real objective of the EU Regulation is the safety of the patient, achieved through a more transparent and balanced customer-manufacturer liaison, the "self-allowance" in EU Regulation looks like a blind spot. Thus, the solution may be regulating tolerance *via* an objective criterion based for instance on the percentage of variation of nominal α associated with ∆T. As this can relate statistically with patient risk in the SPC, in such a scenario tolerance would be a way to let patient safety drive the quality of the product. If so, using manufactured QC would be completely justified by qualitative reliability, productive advantage, and economic fairly.
